# Core temperature and heart rate at the upper limit of the prescriptive zone

**DOI:** 10.14814/phy2.15812

**Published:** 2023-09-09

**Authors:** Thomas E. Bernard, Candi D. Ashley, S. Tony Wolf, W. Larry Kenney

**Affiliations:** ^1^ College of Public Health, University of South Florida Tampa FL USA; ^2^ Exercise Science Program, College of Education University of South Florida Tampa FL USA; ^3^ Department of Kinesiology The Pennsylvania State University University Park PA USA; ^4^ Graduate Program in Physiology The Pennsylvania State University University Park PA USA; ^5^ Present address: Department of Kinesiology University of Georgia Athens GA USA

**Keywords:** heat strain, heat stress, occupational exposure limit, OEL, ULPZ, upper limit of prescriptive zone, WHO report

## Abstract

The expressed goal of limiting workplace heat stress exposures to a core temperature (T_c_) of 38°C traces back to a 1969 World Health Organization Technical Report (WHO Series 412). The actual goal was to limit exposures to the upper limit of the prescriptive zone (ULPZ). To explore the physiological strain at the ULPZ, progressive heat stress protocol data from Penn State University (PSU) and University of South Florida (USF) below and at the ULPZ were used to articulate the relation of *T*_c_ and heart rate (HR) to metabolic rate (MR) with consideration of acclimatization state, clothing, exposure condition (PreULPZ vs. ULPZ), and sex. Regression models demonstrated the association of MR and sex with *T*_c_ and HR. At the ULPZ, women had systematically higher values of *T*_c_ and HR than men at the same MR likely due to higher relative demands. There was no effect for acclimatization state and clothing. As expected for individuals, *T*_c_ was practically constant below the ULPZ and HR exhibited increasing values approaching the ULPZ. At 490 W, the high MR cited in the WHO document, the mean *T*_c_ for men was near the 38°C limit with systematically lower *T*_c_ at lower MRs.

## 1 INTRODUCTION

Occupational recommendations and standards for the prevention of excessive worker heat strain are based on limiting work exposure to a core temperature (*T*_c_) of 38°C for an individual (NIOSH et al., 2016) or for a population average (International Organization for Standardization, 2004a; International Organization for Standardization, 2017) (Note: With regard to terminology, we use a generic *T*_c_ to represent any temperature described as core, deep body, rectal, or gastrointestinal). The origin of the 38°C limit was the WHO Technical Report Series 412 (WHO, 1969) titled “Health Factors Involved in Working Under Conditions of Heat Stress”. After stating the *T*_c_ limit of 38°C for prolonged daily exposures to heavy work, the WHO report follows with guidance for lower *T*_c_ limits at lower metabolic rates (MRs). The intended goal was to limit exposures to the upper limit of the prescriptive zone (ULPZ) proposed (Lind, 1963a) and demonstrated (Lind, 1963b, 1970; Lind et al., 1970) by Lind and promoted by the US National Institute for Occupational Safety and Health (NIOSH) (Dukes‐Dobos & Henschel, 1973). The WHO technical report was less prescriptive for heart rate (HR) allowing a maximum value of 110 bpm with lower values at lower MRs. The panel recognized that there is a HR associated with the “limit of compensable heat stress” (WHO, 1969). Finally, the WHO panel reported on observed sweat rates. The maximum was about 2 L/h for short durations and a note that a 24‐h loss of 12 L would be limiting. Under sustained conditions, sweat rate could be 1 L/h.

While Lind used the ULPZ as a single entity to help describe an occupational exposure limit (OEL), the work‐driven *T*_c_ that he was describing in the prescriptive zone is really a phenomenon assignable to individuals, each of whom has their individual upper limit. This paper uses ULPZ as a value assignable to an individual and not a group.

The 38°C limit has been used in research studies to demonstrate that an individual exposure is above or below an OEL. Further, with the promotion of wearables, direct or inferred measures of *T*_c_ require a limit‐value to function as an alert; and 38°C may be chosen based on that practice. With global warming‐induced increases in worker heat stress and the increasing likelihood that wearables will be used to judge exposures by physiological strain, it is imperative to understand the physiological strain associated with work in the prescriptive zone; that is, at and below the ULPZ.

Articulating the distributions of *T*_c_ and HR at the ULPZ among a healthy, hydrated population will extend the guidance offered in the WHO report (WHO, 1969) and provide context to interpret the observed values. We hypothesized that the physiological strain at the ULPZ as reflected in *T*_c_ or HR is related to MR, and the relationship is influenced by clothing, acclimatization state, and sex.

## 2 METHODS

To examine physiological strain more fully at the ULPZ, data from Penn State University (PSU) (Wolf et al., 2022) and University of South Florida (USF) (Bernard et al., 2005, 2008; Garzón‐Villalba et al., 2017a) were used to interrogate the relation between MR and *T*_c_ and HR. The cited studies were approved by the respective institutional review boards and written informed consent was obtained. Both groups used a progressive heat stress protocol to determine the ULPZ for a given trial for acclimatized participants (USF) and unacclimatized participants (PSU). The typical trial began with climatic conditions that easily allowed thermal equilibrium for the MR and clothing ensemble. A physiological steady state is observed as no changes in *T*_c_ and HR, and typically occurs in the first 30–45 min. Once steady‐state was observed, dry bulb temperature or vapor pressure for PSU or dry bulb at constant relative humidity (RH) for USF were increased in small steps every 5 min, which allowed a quasi‐steady‐state to exist before the next increment. After the critical condition, *T*_c_ increased steadily and continuously. The critical condition was noted using the judgment of experienced investigators as the last climatic condition for which *T*_c_ was steady; and after which *T*_c_ increased about 0.1°C per 5‐min step. The informed judgment method provides the same results as segmented regressions (Wolf et al., 2022). The critical condition represented the ULPZ.

In the PSU studies reported here, participants were tested in a minimally‐clothed ensemble (tee shirt/sports bra, shorts, socks and shoes) in ambient conditions representing a wide range of temperature (33–53°C) and RH (10%–85%) (Cottle, Lichter, et al., 2022; Cottle, Wolf, et al., 2022; Wolf et al., 2022). The participants were healthy, hydrated, and unacclimatized. The MRs were light (Bike Study) and moderate (Walking Study). Metabolic rate was measured twice (at 5 and 60 min) with a real time oxygen uptake system (Parvo Medics TrueOne 2400, Parvo, UT). *T*_c_ and HR data were recorded at the ULPZ and 15 min prior to the ULPZ (PreULPZ).

The USF data were reported previously to examine clothing effects on wet bulb globe temperature (WBGT) at the critical condition (Bernard et al., 2005, 2008). The trials included five clothing ensembles (I_T_ [m^2^ °C W^−1^]/R_e,T_ [m^2^ kPA W^−1^]): Cotton shirt plus trousers (0.19/0.031), Cotton coverall (0.19/0.033), Non‐woven particle barrier (0.19/0.035), Water barrier using microporous film (0.19/0.041), and Vapor barrier (0.19/0.084). Metabolic rate was measured using a 3‐min Douglas bag sample at 30, 60 and 90 min into the trial. In one study, MRs were considered at three levels (Low, Moderate, and High) in which each participant wore all five clothing ensembles at all three MRs (Met Study) with RH held at 50%. A second study (RH Study) used the five clothing ensembles at three humidity levels (20%, 50%, and 70% RH) and moderate MR. *T*_c_ and HR data at the ULPZ (i.e., critical conditions) were reported for acclimatized participants (Ashley et al., 2008; Garzón‐Villalba et al., 2017b). *T*_c_ and HR were also noted for each trial 15 min prior to the critical condition for an observation about 3°C‐WBGT below the ULPZ (Garzón‐Villalba et al., 2017b), called here the PreULPZ. For analysis, two ensembles (cotton shirt plus trousers and cotton coverall) were combined into one ensemble called woven clothing because no significant difference in thermal characteristics was found between them (Bernard et al., 2005, 2008; Caravello et al., 2008).

Table 1 describes the participants for each of the four studies (Bike, Walk, Met, and RH) divided into men and women. The characteristics were reported as the mean and standard deviation of age, height, weight, and body surface area. For the two PSU studies, most of the participants completed two trials for Bike and Walk. The two studies from USF had fewer participants than PSU, but each participant contributed about 15 trials rather than 4. Table 2 describes the number of trials for each study along with the mean and standard deviation for MR, *T*_c_, and HR at the ULPZ. The standard deviations for USF Met study were higher than the other trials due to the design, which called for three levels of MR.

**TABLE 1:** Distribution of participant characteristics (age, weight, height, and body surface area) by study.

			Age [year]	Weight [kg]	Height [m]	Body surface area [m^2^]
Study^a^	Sex	N	Mean ± SD	Mean ± SD	Mean ± SD	Mean ± SD
PSU^b^	Men	24	23.7 ± 4.2	84.7 ± 15.4	1.81 ± 0.08	2.05 ± 0.18
Bike	Women	23	23.0 ± 3.8	68.2 ± 15.2	1.65 ± 0.05	1.75 ± 0.17
PSU^b^	Men	23	23.3 ± 3.8	83.0 ± 13.2	1.81 ± 0.07	2.03 ± 0.16
Walk	Women	26	22.6 ± 3.7	68.1 ± 15.0	1.66 ± 0.05	1.75 ± 0.18
USF^c^	Men	12	27.3 ± 9.4	84.5 ± 14.4	1.76 ± 0.11	2.01 ± 0.20
Met	Women	4	23.0 ± 4.7	64.2 ± 18.0	1.65 ± 0.06	1.70 ± 0.22
USF^c^	Men	9	29.2 ± 6.8	97.4 ± 18.4	1.83 ± 0.05	2.19 ± 0.20
RH	Women	4	34.0 ± 8.9	63.5 ± 20.0	1.63 ± 0.06	1.68 ± 0.26

^a^ Study: PSU Bike (light metabolic rate); PSU Walk (moderate metabolic rate); USF Met Trials (low, moderate and high metabolic rates at 50% relative humidity); USF RH Trials (20, 50, 70% relative humidity at moderate metabolic rate).

^b^ The PSU studies used more participants and each participant completed about two trials in each study.

^c^ The USF studies used fewer participants and each participant completed about 15 trials.

**TABLE 2:** Distribution of progressive heat stress trials by the four types of trials and clothing for the mean ± standard deviation of metabolic rate (MR), core temperature (*T*_c_) and heart rate (HR) at the upper limit of the prescriptive zone (ULPZ); and the distributions by sex for the four types of trials.

				MR [W]	*T*_c_ [°C]	HR [bpm]
	Study^a^	Clothing	Number of trials^b^	Mean ± SD	Mean ± SD	Mean ± SD
PSU	Bike	SC	91	161 ± 37	37.38 ± 0.27	94 ± 14
	Walk	SC	99	280 ± 57	37.66 ± 0.35	113 ± 21
USF	Met Trials	WC	90	347 ± 118	37.67 ± 0.30	117 ± 20
		PB	75	330 ± 102	37.69 ± 0.35	116 ± 17
		WB	45	346 ± 126	37.71 ± 0.33	119 ± 19
		VB	49	342 ± 123	37.77 ± 0.37	124 ± 21
	RH Trials	WC	86	305 ± 82	37.77 ± 0.36	117 ± 16
		PB	44	314 ± 91	37.76 ± 0.25	114 ± 13
		WB	46	302 ± 84	37.78 ± 0.29	116 ± 14
		VB	45	328 ± 74	37.64 ± 0.33	112 ± 17

		Sex				
PSU	Bike	Men	49	183 ± 31	37.26 ± 0.22	91 ± 11
		Women	42	135 ± 24	37.51 ± 0.25	97 ± 16
	Walk	Men	52	314 ± 47	37.54 ± 0.34	105 ± 16
		Women	47	243 ± 42	37.79 ± 0.31	121 ± 21
USF	Met Trials	Men	189	361 ± 114	37.71 ± 0.35	115 ± 17
		Women	70	286 ± 103	37.67 ± 0.30	127 ± 22
	RH Trials	Men	143	348 ± 67	37.68 ± 0.30	110 ± 15
		Women	78	242 ± 63	37.86 ± 0.33	124 ± 12

Abbreviations: PB, nonwoven particle barrier; SC, semi‐clothed; VP, vapor barrier; WB, microporous water barrier; WC, cotton shirt/trousers or cotton coverall.

^a^ Study: Bike (light metabolic rate); Walk (moderate MR); Met Trials (low, moderate and high metabolic rates at 50% relative humidity); RH Trials (20, 50, 70% relative humidity at moderate metabolic rate).

^b^ Note that there was considerable overlap in participants between the two PSU studies with each participant completing about two trials in each study. The USF studies used fewer participants and each participant completed about 15 trials (5 clothing levels at three metabolic rates or three relative humidity levels).

### 2.1 Data analysis

JMP 16 (SAS, Cary NC) was used for data analysis. The first step was to assess the effects of clothing using the USF data. A 4‐way mixed effects ANOVA with MR as a continuous variable; clothing ensembles (4 levels), and sex as fixed effects plus the interaction of MR and sex, and participants as a random effect was used to look for clothing effects on *T*_c_, and HR, while accounting for other treatment effects.

If there were no clothing effects, *T*_c_ and HR can be examined with the following model that included MR (MR, continuous), sex (Sex: M‐men and W‐women), acclimatization state (Accl: UN‐unacclimatized and A‐acclimatized), Exposure Condition (Cond: PreULPZ and ULPZ), and the interaction of MR and sex (MR × Sex) (Equation 1). In Equation 1, y represented one of the dependent variables (*T*_c_ or HR).

(1) $$\begin{array}{c} y=\alpha +\beta_{1}\;\mathrm{MR}+\beta_{2}\;\mathrm{Sex}+\beta_{3}\;\text{Accl}+\beta_{4}\;\text{Cond}\\ \;+\beta_{5}\;\mathrm{MR}\times \mathrm{Sex}+\varepsilon \end{array}$$

The data analysis was performed in two parts. Part A was performed first. The model (Equation 1) was used in a forward stepwise linear regression looking for the minimum Bayesian information criterion (BIC), which balances fit with complexity. The important contributors were identified at the minimum BIC. Using the minimum BIC allowed for the elimination of factors that did not contribute meaningfully to explaining overall error.

In Part B, the important contributors were used in a least squares linear regression with the addition of participants as a random effect. The final regression model used the significant contributors at *α* < 0.05. The error term was reported as the root mean square error (RMSE).

## 3 RESULTS

### 3.1 Clothing

Based on a 4‐way mixed effects ANOVA, there was not a significant effect of clothing for *T*_c_ (*p* = 0.31) or HR (*p* = 0.34). There were two important differences between the PSU and USF data; the PSU studies were performed in a minimally clothed state (e.g., tee shirt and shorts) and the participants were unacclimatized. Given that there were no differences over a large range of evaporative resistances in the USF clothing, we assumed that lack of effect carried over to the PSU clothing. Thus, any difference between the datasets was assigned to acclimatization state.

### 3.2 Core temperature

In the stepwise forward regression model, MR, sex, acclimatization, and exposure condition were all important contributors to *T*_c_ (*p* < 0.001) and the interaction did not lower the BIC (see Table 3, Part A). In the least squares regression (Table 3, Part B), only MR, sex and exposure condition were significant, and acclimatization (*p* = 0.12) was not. The difference in *T*_c_ for exposure condition (i.e., between PreULPZ and ULPZ) was relatively small (0.04°C). The model was further reduced to *T*_c_ at the ULPZ (*T*_c,ULPZ_) and expressed as (Equation 2)

(2) $$\begin{array}{c} T_{c,\text{ULPZ}}=37.2+0.14\;\left(1\;\text{if women};,–1\;\text{if}\;\mathrm{men}\right)\\ \;+0.0015\;\mathrm{MR}\left[W\right]\pm 0.24{}^{\circ}C \end{array}$$

**TABLE 3:** Report of important contributors from forward stepwise regression using minimum BIC (Part A) and for linear regression with coefficients starting with important contributors from Part A and adding participants as random effect (Part B).

Part A: Stepwise forward regression with important contributors, level of significance, and coefficient with root mean square error (±RMSE)						
Stepwise regression	Metabolic rate (MR)	Sex (if men)	Acclimatization state (if Unacclimatized)	Exposure condition (if PreULPZ)	MR × Sex (if men)	Intercept ± RMSE
*T*_c_	*p* < 0.001 0.00124	*p* < 0.001 −0.117	*p* < 0.001 −0.0410	*p* < 0.001 −0.0360	not entered	37.27 ±0.31
HR	*p* < 0.001 0.0771	*p* < 0.001 −9.69	*p* < 0.001 −2.71	*p* < 0.001 −4.48	*p* < 0.001 −0.0185 (MR‐298)	88.4 ±16.0

Part B: Final Regression with participants as a random effect. Reporting level of significance, coefficient and ± standard error, as well as *r* ^2^ and root mean square error (±RMSE).							
Final regression	Metabolic Rate (MR)	Sex (If women)	Acclimatization state	Exposure condition (If PreULPZ)	MR × Sex (If women)	Intercept	*r* ^2^ ± RMSE
*T*_c_	*p* < 0.001 0.00155 ±0.00008	*p* < 0.001 0.140 ±0.025	ns (*p* = 0.12) removed	*p* < 0.001 −0.036 ±0.006	–	37.18 ± 0.03	0.57 ± 0.23
HR	*p* < 0.001 0.102 ±0.004	*p* < 0.001 11.2 ±1.6	ns (*p* = 0.20) removed	*p* < 0.001 −4.5 ± 0.3	*p* < 0.001 −0.0269 (MR‐298) ± 0.0040	81.6 ± 1.8	0.75 ± 9.9

*Note*: not included in Part B analysis (not identified as an important contributor in Part A, stepwise regression); ns: not significant (α = 0.05) in Part B analysis. In Part B, there are logical statements for two dichotomous factors (Sex and Exposure Condition) and the interaction (Sex and MR) where the term is included in the regression for Women and for PreULPZ.

The equation contains a logical test for men and women. MR[W] is MR in Watts. The value after ± sign is the RMSE.

**FIGURE 1:**
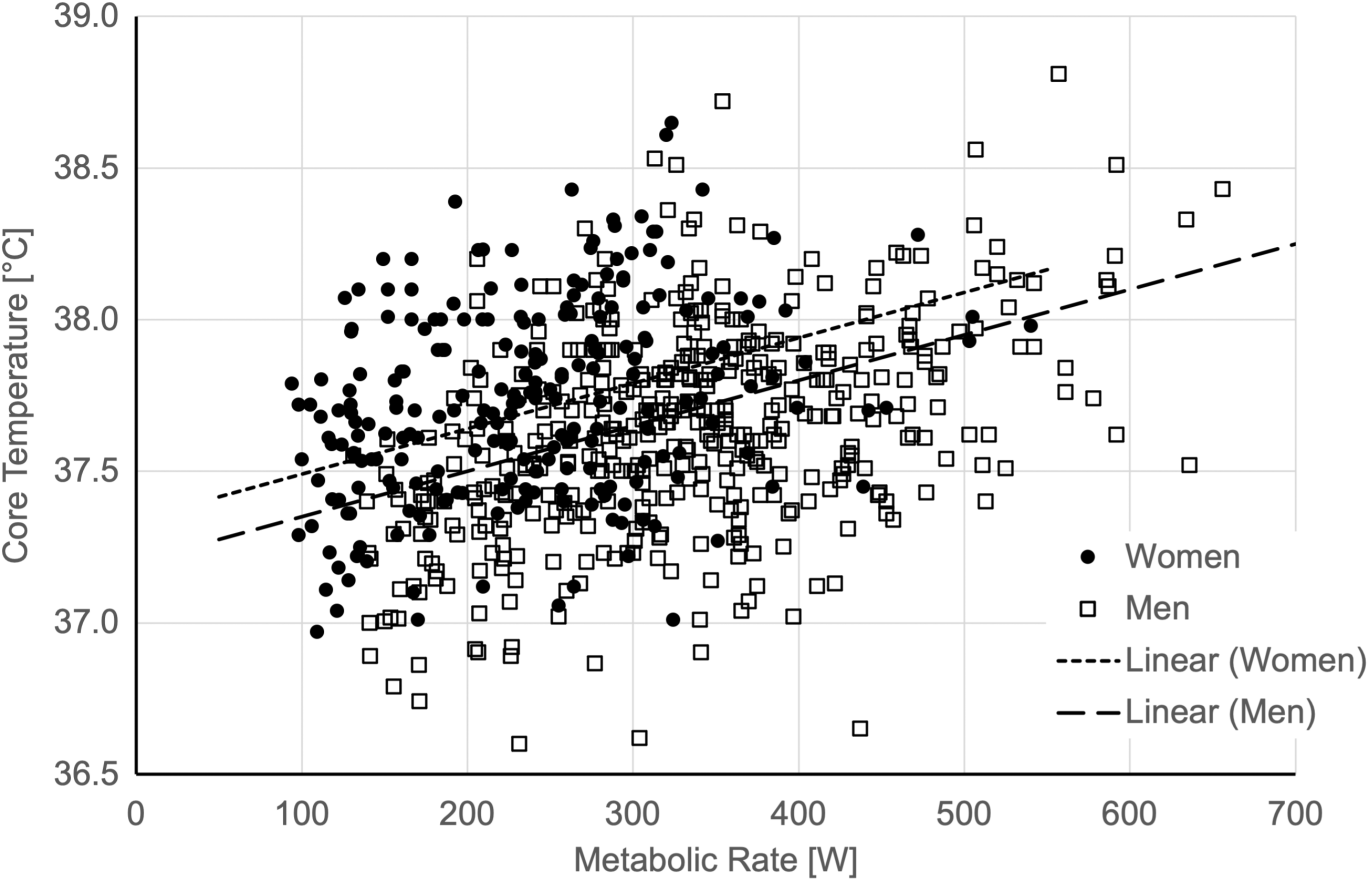
Relationships between MR [W] and core temperature [°C] at the ULPZ for men and women separately (Equation 2).

Figure 1 summarizes the *T*_c,ULPZ_ data and regression lines for men and women at the ULPZ.

### 3.3 Heart rate

The BIC‐optimized stepwise forward regression model for HR indicated that the important contributors were MR, sex, acclimatization state, exposure condition and the interaction of MR and sex (all *p* < 0.001; Table 3 Part A). In the final linear regression with participants as a random effect (Table 3, Part B), there were significant contributions from MR, sex, and exposure condition as well as the interaction of MR and sex (*p* < 0.001). Acclimatization state was not a significant contributor (*p* = 0.20).

Based on the Part B linear regression, the difference between PreULPZ and ULPZ was 4.5 bpm higher for HR at the ULPZ. The next step was to consider only the HR at the ULPZ (HR_ULPZ_). The final model for HR_ULPZ_ is provided as Equation 3.

(3) $$\begin{array}{c} {\mathrm{HR}}_{\text{ULPZ}}=86+10\;\left(1\;\text{if women};,–1\;\text{if}\;\mathrm{men}\right)\\ \;+0.027\;\left(\mathrm{MR}\left[W\right]–298\right)\;\left(1\;\text{if women};,–1\;\text{if}\;\mathrm{men}\right)\\ \;+0.097\;\mathrm{MR}\left[W\right]\pm 10\;\mathrm{bpm} \end{array}$$

The equation contains a logical test for men and women. MR[W] is MR in Watts. The third term in the equation represents the interaction of sex and MR. The interaction term changes both the slope and the intercept from men to women. The value after ± sign is the RMSE.

**FIGURE 2:**
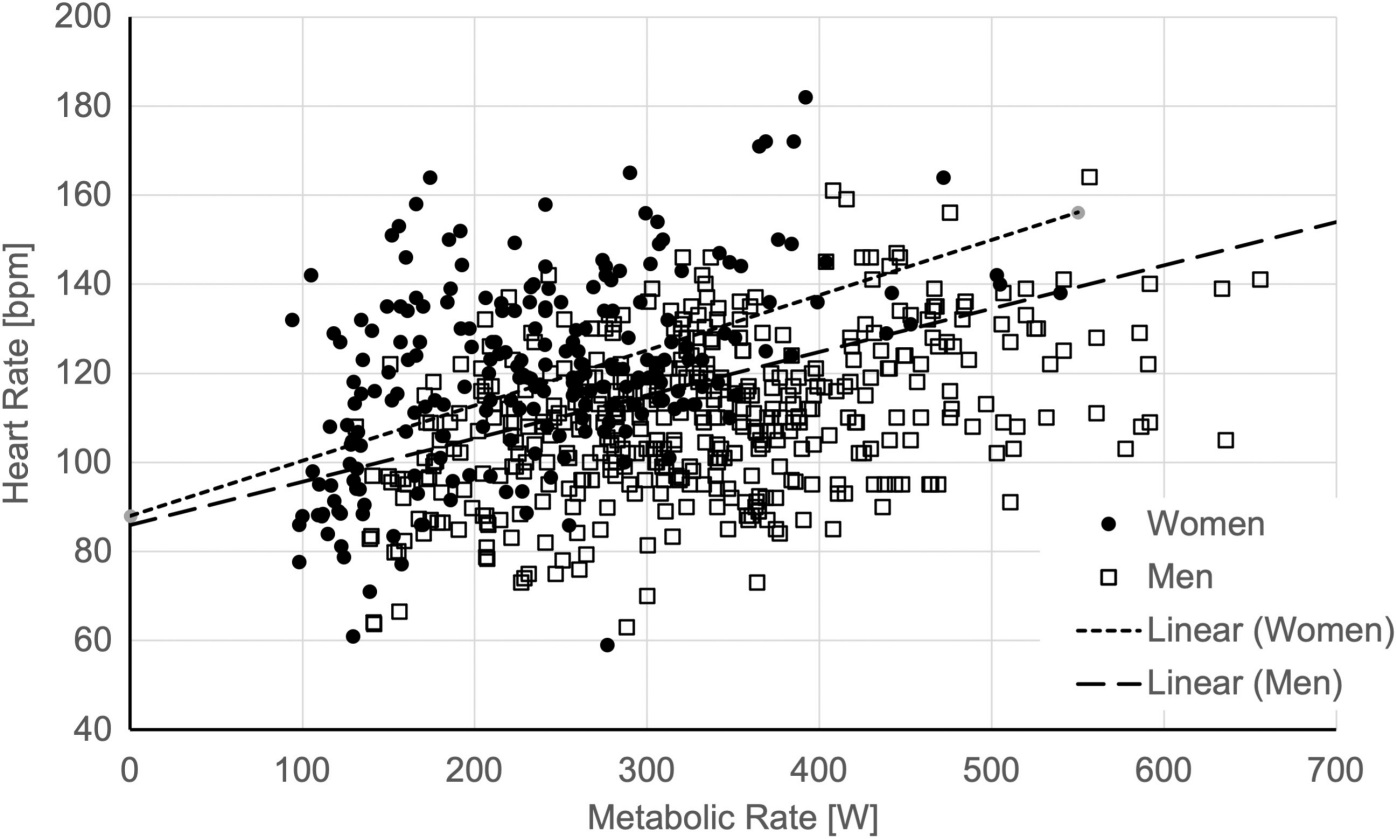
Relationships between MR [W] and heart rate [bpm] at the ULPZ for men and women (Equation 3).

Figure 2 presents the HR data and Equation 3 for men and women at the ULPZ.

## 4 DISCUSSION

Although maintaining the ability of a worker to stay in thermal balance (i.e., in the prescriptive zone) is the primary objective of WBGT‐based heat stress recommendations and standards, a threshold *T*_c_ of 38°C is often associated with those standards. The present study leveraged data collected on over 75 participants across two universities to describe physiological strain at the ULPZ for young (generally below 30), healthy, hydrated men and women using a progressive heat stress protocol. The studies included a range of MRs, environmental conditions, and clothing ensembles as well as acclimatization state and both sexes.

The clothing ensembles used in the USF studies included woven clothing, non‐woven particle barrier, a microporous water barrier, and a vapor barrier with a range of total evaporative resistances from 0.031 to 0 0.084 m^2^ kPa W^−1^. No difference in *T*_c_ and HR at the ULPZ was found among these ensembles. While the environments at the ULPZ were different (Bernard et al., 2005, 2008), the physiological strain represented by *T*_c_ and HR was similar. The extension of no differences in strain to the semi‐clothed ensemble of the PSU studies was reasonable. By assigning no‐effect for clothing, differences between the PSU and USF populations were assigned to acclimatization state.

### 4.1 Core temperature

The WBGT‐based OELs were designed to be protective of most healthy, hydrated people (Garzón‐Villalba et al., 2017a). Virtually all of the individual WBGT values of the ULPZ at a given MR and standard woven clothing were above the OEL. The combined PSU and USF data included exposures greater than the OEL to capture ULPZ data for a range of heat tolerance rather than only at the low end of the distribution. There was little difference (0.04°C) in *T*_c_ between PreULPZ and ULPZ data, thus confirming that an individual's *T*_c_ was approximately the same independent of environment at exposures below or at the ULPZ. This independence of *T*_c_ with environment was reported by Lind (Lind, 1963a) and in the WHO report (WHO, 1969); and was Lind's rationale for the prescriptive zone.

In Equation 3 and Figure 1, the line for women was higher than for men. This might be due to systematic differences in maximal aerobic capacity (Loe et al., 2013) and the fact that work‐driven *T*_c_ (those in the prescriptive zone) is better explained as a percent of maximal aerobic capacity (Saltin & Hermansen, 1966).

The 38°C limit for heavy work recommended by the WHO was likely based on the highest workload in Lind's presentation of the ULPZ, which was 490 W (Lind, 1963a). The data in Figure 1 at 490 W provided a picture of population distribution. The population mean for men (point on the regression line) was approximately 38°C, which confirms the value in the WHO report (WHO, 1969). It was also clear that many observations would be above 38°C. Thus, the value of 38°C does not have any utility for demonstrating exposures below the ULPZ at 490 W for an individual. ISO and ACGIH also suggest a *T*_c_ limit of 38.5°C in case of individual strain monitoring (ACGIH®, 2023; International Organization for Standardization, 2004b). This higher limit does not appear to be associated with a sustainable exposure but rather for exposures when thermal equilibrium cannot be sustained.

With regard to our hypothesis, *T*_c_ at the ULPZ was clearly influenced by MR and sex, but not clothing and acclimatization state. The effect of sex may be due to the consideration of absolute MR and not a workload normalized for aerobic capacity. As the MR increases, the proportion of individuals with *T*_c_ higher than 38°C increases.

The WBGT exposure limits as a function of MR derive from the Lind's vision of the UPLZ (Dukes‐Dobos & Henschel, 1973). Rather than being distracted by a *T*_c_, the purpose of the WBGT‐based OEL was to limit exposures at or below the ULPZ for most people. To demonstrate effectiveness, the exposures should lead to compensable heat stress and not to a specific value of *T*_c_. Garzón and colleagues have shown the difficulty of using *T*_c_ to categorize an exposure as being above or below the ULPZ (or in the parlance of their paper, sustainable or unsustainable) even adjusting for MR (Garzón‐Villalba et al., 2017b). In this context, our data confirmed that a single, fixed *T*_c_ threshold limit does not provide useful insight into whether the exposure is below or above the ULPZ for an individual.

### 4.2 Heart rate

Looking at the HR data, it was clear that there was an effect due to MR, exposure condition (PreULPZ vs. ULPZ) and sex. The difference for exposure condition was not surprising because HR increases typically precede the increase in *T*_c_ to meet the increasing need to dissipate heat. This cardiovascular adjustment above that dedicated to supporting resting and work metabolic demands was also anticipated in the WHO report (WHO, 1969) and described as thermal cardiac reactivity (Kampmann et al., 2001) and as the elevation of heart rate of thermal origin (∆HR_T_) in ISO standards (International Organization for Standaization, 2004b; International Organization for Standaization, 2021). The ULPZ data do show that in the early stages of thermal cardiac reactivity; that is, as the exposure is approaching ULPZ, the HR increases without a change in *T*_c_. The mean difference between the PreULPZ and ULPZ points were on the order of 10 bpm. As with *T*_c_, there was a difference in HR at the ULPZ due to sex, wherein women exhibited a steeper slope resulting in an increasing difference as MR increased. Because the independent variable was absolute MR, the women on average were working at a higher percent of their maximal aerobic capacity and thus required a greater cardiovascular response.

With regard to our hypothesis, the HR at the ULPZ depended on MR, sex as a fixed effect and as an interaction with MR, but not clothing or acclimatization state. The mean response for HR values associated with the ULPZ is described by Equation 3 and the overall picture is provided in Figure 2. Similar to the findings of Garzón‐Villalba, et al. (2017b), the profile of HR responses in the current study was not effective for demonstrating whether an exposure is below the ULPZ.

### 4.3 Comments and limitations

USF investigators have described the progressive heat stress protocol as a method to identify a critical threshold below which thermal equilibrium could be established with constant *T*_c_ (Bernard et al., 2005, 2008; O'Connor & Bernard, 1999), and asserting that thermal equilibrium could not be maintained above the critical condition. In retrospect, a more precise statement would be that the critical threshold represented the ULPZ for the individual under the exposure conditions and that for some heat stress conditions just above the critical conditions thermal equilibrium could be maintained, albeit at a higher *T*_c_ (Lind, 1963a; Pandolf & Burr, 2001).

The work‐specific *T*_c_ and HR are best described as a function of relative aerobic capacity (Pandolf & Burr, 2001; Saltin & Hermansen, 1966). When the maximal aerobic capacity is not known, the relationships described in this paper provide values at the ULPZ for healthy, hydrated individuals regardless of acclimatization state. Because the MRs were expressed in absolute terms, the women as a group were working at a higher percent of their maximal aerobic capacity (Loe et al., 2013) and thus had higher values of *T*_c_ compared to men.

Both the ACGIH (ACGIH, 2023) and ISO (International Organization for Standardization, 2004b) suggest a *T*_c_ limit of 38.5°C, but this limit appears to be a safe limit for individuals who may not be in thermal equilibrium. That is especially true in the context of the data reported here.

Because the progressive heat stress protocol has short steps, it is difficult to assess the sweat rate at the critical condition. This limitation does not allow any comment on the WHO discussion of sweat rate. It is worth noting the ISO Predicted Heat Strain does address sweat rate and sweat volume limits (International Standards Organization, 2004a).

The WHO report states that exposures to heat stress in the prescriptive zone up to the ULPZ could be sustained during a normal working day. This may not be the case. An unpublished analysis of Kuhlmeier's data for 1‐h exposures (Kuhlemeier et al., 1976) showed a similar distribution of *T*_c_ as presented in Figure 1. Some investigators have demonstrated a steady increase in *T*_c_ in some individuals over a 2‐h continuous exposure at the OEL, which should be below individual values of ULPZ for most healthy and hydrated participants (Gagnon & Kenny, 2011; Kaltsatou et al., 2020; Lamarche et al., 2017; Meade et al., 2016). Lind (Lind, 1970) reported mean values for *T*_c_ and HR at 1‐h intervals during 3‐h exposures at four levels of heat stress (see his figure 4). The lower two exposures were at and below his proposed exposure limits. Average *T*_c_ rose from about 38.1°C at the first hour to 38.2°C in the second and third hours, indicating a small drift. Average HR showed a steady increase of 15 bpm over the 3 h, indicating an increasing strain with duration.

The major differences between the PSU and USF data were clothing and acclimatization state. The argument that clothing was not important was based on the relatively small extrapolation to semi‐clothed from a range of clothing that included woven cotton to vapor barrier. Because there was no significant effect for acclimatization in the final (Part B) models, these findings suggest that there was no difference between PSU and USF data whether it was acclimatization, clothing, or some other unaccounted‐for effects. That is, the assumption about clothing was not central to the findings.

Another difference between the two datasets was the range of MRs. The span of MRs was somewhat different between the two datasets (see Table 2). The PSU data included light work (Bike with mean MR of 160 W) and moderate work (Walking at an average rate of 280 W). The mean MR for the two USF datasets were about 340 W for Met Trials and 310 W for the RH Trials; and the Met Trials included MRs above 400 W. Following the argument in the previous paragraph, these differences did not appear to affect the results based on the lack of findings for acclimatization state, which was the surrogate metric to represent the differences between the datasets from the two universities. In this context, the level of significance for acclimatization on *T*_c_ (*p* = 0.09) is not a strong finding. There may be an effect that would be better explored by comparing the differences for the same person before and after acclimatization.

Limitations to the use of physiological markers to demonstrate exposures below the ULPZ are significant. This paper demonstrates that there is sufficient variability that makes recommending a clear limit difficult. This difficulty is confounded by how well the MR is known. For instance, in laboratory studies it makes a difference if an average MR is used to represent the exposure or if individual values are used. In the field, estimated MRs have more uncertainty (International Organization for Standardization, 2021). There is further uncertainty if the physiological limits are applicable to conditions that have existed for hours.

## 5 CONCLUSIONS

The main conclusion was that heat stress below the ULPZ for most individuals, which is the basis of the WBGT occupational exposure assessments, was not associated with a fixed value of *T*_c_ and thus a value of 38°C cannot be used to judge whether an exposure is compensable or not. The combined data from USF and PSU supported expectations that steady‐state *T*_c_ would exceed 38°C for some individuals with MRs near 200 W, and the portion of individuals that would exceed 38°C increased steadily above 200 W. Average values above 38°C for a population of workers were most likely to occur above 500 W (see Figure 1).

A second conclusion addressed the intention that OELs based on the ULPZ (ACGIH®, 2023; International Organization for Standardization, 2017; NIOSH et al., 2016) maintain *T*_c_ below 38°C in accordance with WHO recommendations. While the goal may be a fixed *T*_c_, there is no support for treating a fixed *T*_c_ as the determinant for meeting the practical intentions of ACGIH, NIOSH and WHO to keep exposures in the compensable heat stress range for most people. To determine acceptable exposures, the criterion should be to demonstrate that the exposure is compensable for most individuals. A corollary of this conclusion is the need to 1) evaluate the heat stress for each individual, 2) determine if the exposure is above or below the OEL, and 3) assess whether there is evidence of compensable or uncompensable heat stress for the population to test the proposition that most are protected (Bernard et al., 2022).

## DISCLAIMERS

The authors declare no conflict of interest relating to the material presented in this article. Its contents, including any opinions and/or conclusions expressed, are solely those of the authors.

One of the authors [Bernard] has acted as an expert witness for both private companies and OSHA in litigation concerning heat stress exposures and may in future serve as an expert witness in court proceedings related to heat stress.

## Data Availability

All reasonable requests for data will be honored.
